# Supplementary material for: Individual and Environmental Factors Associated with Participation in Physical Activity as Adolescents Transition to Secondary School: A Qualitative Inquiry
Source: Int J Environ Res Public Health. 2020 Oct 20;17(20):7646. doi: 10.3390/ijerph17207646 (PMC7588993; doi:10.3390/ijerph17207646)
Supplement: Supplementary file 1 [file ijerph-17-07646-s001.pdf]

PARENT INTERVIEW SCRIPT

1-YR PROSPECTIVE INTERVIEW PROTOCOL IN GRADE 8

[*Test and set up audio recorders. Upon arrival, provide brief overview of what the study is about (refresher of consent/assent) have participant verify all contact information and child's new secondary school.*)

INTRODUCTORY SCRIPT

Thank you for taking the time to participate in our study. The purpose of this interview is to understand how your child's physical activity, sedentary activity (meaning time spent in front of a screen) and eating habits changed between grade 7 and grade 8. We are also interested in what factors you think influence your child's current activity and eating habits or the change in their activity and/or eating habits (if any change occurred). This interview will take about 45-60 minutes and you will receive $35.

Do you have any questions?

INTERVIEW SCRIPT

Ok then – let's get started.

I will be recording the interview because I don't want to miss any of your comments or thoughts. What you say will only be shared with the research team. Also, we ensure that any information you tell me today will not identify you. You can end the interview or turn off the recorder at any time. Do you have any questions? Can I begin recording our interview?

> *If Yes – proceed otherwise stop the interview and thank the participant for coming to the interview.*

[*Turn on tape recorder – record the date and time of the interview prior to starting the interview*]

Physical Activity

1. Generally speaking, how active is your child right now in terms of physical activities or sports (inside of school/ outside of school)? Can you provide me an idea of what your child does in a typical week?
   - Has your child's physical activity levels changed since s/he was in Grade 7?
   - Why do you think your child is more/less active than before?

2. Are there things that you do as a parent that influences your child's physical activity?
   - Has this changed since Grade 7?
   - Have your expectations changed since your child was in Grade 7?
   - How has it changed?
   - Why do think that is?

3. Are there other things at home or around your home that make it easier or harder for your child to be active?
   - Has this changed since Grade 7?
   - How has it changed?
   - Why do think that is?

4. How does your child typically get to and from school? How often does he/she [mode of transportation] to and from school?
   - Has this changed since Grade 7?
   - How has it changed?
   - Why do think that is?

5. Are there things at school and around school that you think influence how much time your child spends being active?
   - Has this changed since Grade 7?
   - How has it changed?
   - Why do think that is?

6. Do you think your child's friends or sibling(s) influence how much time your child spends being active?
   - Has this changed since Grade 7?
   - How has it changed?
   - Why do think that is?

7. Are there other things that influence how much time your child spends being active?
   - Has this changed since Grade 7?
   - How has it changed?
   - Why do you think that is?

8. So out of all the things we talked about. We talked about the influence of the home, school, friends and environment on your child's physical activity. How would you rank these influences in terms of which one is most important all the way down to least important? You can start with the most important.
   - Has this changed since Grade 7?
   - How has it changed?
   - Why do think that is?

Probes: What do you do to help your child be active? How important is PA to you? Once they have explained the sports their child participates in, ask the following—How did your child end up participating in these activities? Is this something you came up with or your child? What about play during recess or lunch?

General

1. We talked about how you as a parent influence your child's physical activity. Can you comment on whether the things you do as a parent, in terms of your expectations or what you do in general, is different or the same as the "mother/father" of your child?
   - Has this changed since Grade 7?
   - How has it changed?
   - Why do think that is?

2. Is there anything else you wanted to share with us?

We are at the end of the interview. [*Turn off audio recorder*]
Thank you again for taking the time to speak with me today. I really appreciate your participation. If this interview raises any thoughts in the coming days, please feel free to contact me. As a token of appreciation, I am providing you with $35 to compensate you for your time.

TEEN INTERVIEW SCRIPT

1-YR PROSPECTIVE INTERVIEW PROTOCOL FOR GRADE 8

[*Test and set up audio recorders. Upon arrival, provide brief overview of what the study is about (refresher of consent/assent) have participant verify all contact information and ask about child's new secondary school.*)

INTRODUCTORY SCRIPT

Thank you for taking the time to participate in our study. The purpose of this interview is to understand how your physical activity, screen time and eating habits have changed between grades 7 and 8. As well, we are interested in what factors you think have influenced your activity and eating behaviours during this transition. This interview will take about 45-60 minutes and you will receive $35 for participating.

Do you have any questions?

INTERVIEW SCRIPT

Ok then – let's get started.
I will be recording the interview because I don't want to miss any of your comments or thoughts. What you say will only be shared with the research team. Also, we ensure that any information you tell me today will not identify you. You can end the interview or turn off the recorder at any time. Do you have any questions? Can I begin recording our interview?

If Yes – proceed otherwise stop the interview and thank the participant for coming to the interview.
[*Turn on tape recorder – record the date and time of the interview prior to starting the interview]*

Physical Activity

1. How active are you right now in terms of physical activities or sports (inside of school/ outside of school)? Can you provide me an idea of what you do in a typical week?
    - Have your physical activity levels changed since you were in Grade 7?
    - Why do you think you are more/less active than before?

2. Are there things that your parents do that influences (opportunities or expectations) your physical activity?
    - Has this changed since Grade 7?
    - Have their expectations changed since you were in Grade 7?
    - How have they changed?
    - Why do you think that is?

3. Are there other things (places) at home or around your home that make it easier or harder for you to be active?
    - Has this changed since Grade 7?
    - How have they changed?
    - Why do you think that is?

4. How do you typically get to and from school? How often do you [mode of transportation] to and from school?
    - Has this changed since Grade 7?
    - If driven as whether it is possible to walk
    - Why do you think that is?

5. Are there things at school or around school that influence how much time you spend being active?
    - Has this changed since Grade 7?
    - How have they changed?
    - Why do you think that is?

6. You changed school, as your network of friends changed? Use the answer to ask whether new or old friend are more or less active than last year and whether they are interested in PA? Do you think your friends or sibling(s) influence how much time you spend being active?
    - Has this changed since Grade 7?
    - How have they changed?
    - Why do you think that is?

7. Are there other things that influence how much time you spend being active?
    - Has this changed since Grade 7?
    - How have they changed?
    - Why do you think that is?

8. So out of all the things we talked about. We talked about the influence of the home, school, friends and environment on your physical activity. How would you rank these influences in

terms of which one is most important all the way down to least important? You can start with the most important.

- Has this changed since Grade 7?
- How have they changed?
- Why do you think that is?

General

1. We talk about how your parents influence your physical activity. Can you comment on whether the things your mother and father do, in terms of their expectations or what they do in general, are different or the same?
    - Has this changed since Grade 7?
    - How has it changed?
    - Why do think that is?

2. Is there anything else you wanted to share with us?

We are at the end of the interview. [*Turn off audio recorder*]

Thank you again for taking the time to speak with me today. I really appreciate your participation. If this interview raises any thoughts in the coming days, please feel free to contact me. As a token of appreciation, I am providing you with $35 to compensate you for your time.
